# Electrochromic Type E-Paper Using Poly(1H-Thieno[3,4-d]Imidazol-2(3H)-One) Derivatives by a Novel Printing Fabrication Process

**DOI:** 10.3390/ma4122171

**Published:** 2011-12-14

**Authors:** Yoshiro Kondo, Hirofumi Tanabe, Hidetoshi Kudo, Kirihiro Nakano, Tomiaki Otake

**Affiliations:** 1 Kuraray America, Inc., 11500 Bay Area Boulevard, Pasadena, TX 77507, USA; 2 Tsukuba Research Center, Kuraray Co. Ltd., 41 Miyukigaoka, Tsukuba, Ibaraki 305-0841, Japan; E-Mails: hirofumi_tanabe@kuraray.co.jp (H.T.); hidetoshi_kudo@kuraray.co.jp (H.K.); kirihiro_nakano@kuraray.co.jp (K.N.); tomiaki_otake@kuraray.co.jp (T.O.)

**Keywords:** electronic papers, conductive polymers, printable electronics

## Abstract

In this study, we report poly(1H-thieno[3,4-d]imidazol-2(3H)-one) (pTIO) derivatives for an electrochromic (EC) type e-paper and its novel printing fabrication process. pTIO is a kind of conductive polymer (CP) s which are known as one of the EC materials. The electrochromism of pTIO is unique, because its color in doped state is almost transparent (pale gray). A transparent state is required to show a white color in a see-through view of an EC type e-paper. An electrochromism of CP has a good memory effect which is applicable for e-paper. The corresponding monomers of CP are able to be polymerized with an electrochemical method, which be made good use of for the fabrication process of e-paper. pTIO derivatives are copolymerized with other pi-conjugated X unit, which adjusts the color of electrochromism. Finally, we fabricated a segment matrix EC display using pTIO derivatives by ink-jet printing.

## 1. Introduction 

E-paper is an interesting area for electronics, because it can be substituted for printed papers, which enable a better life for humans by solving the problems concerning environmental issues such as exhaustion of natural resources due to the paper productions. In addition, the flexible and reflective characteristics of the device can open up new concept applications [1].

Various techniques have been made to try to realize the practical usage of E-paper, such as electrophoresis type [2], electro-wetting type [3], microelectromechanical mirror type [4], reflective liquid crystal type and electrochromic type [5].

Electrochromic(EC) type has various advantages such as low voltage, high reflectance and a variety of structure designs, as examples, parallel or stack structure. There are some kinds of EC materials, which are inorganic (metal oxide) type, for example WO_3_[6], organic molecular type, for example Viologen [7], polymer type, for example conductive polymer (CP) [8], and organic/inorganic hybrid type [9]. 

**Figure 1:**
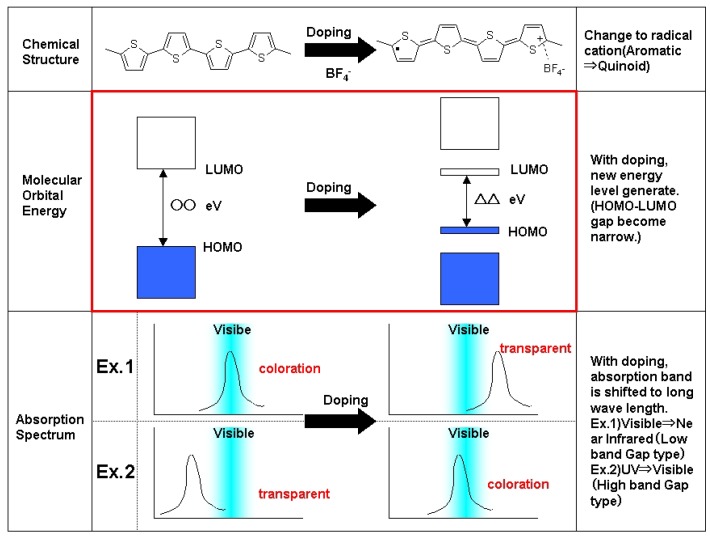
Principle of polymer electrochromism.

CP has some features. One is electrochromism. The principle of polymer electrochromism is shown in Figure 1. CP has two states; the doping state and the de-doping state. The structure of CP in the doping state is called a quinoidal structure meanwhile that in the de-doping state is called an aromatic structure. Usually, the de-doping state of CP is not conductive and has an absorption band in a visible area. This absorption band is derived from band gap energy. When CP is applied voltage, CP donates (or accepts) an electron and is transformed to a quinoidal structure. CP in a quinoidal structure is conductive. Counterpart ions (anions or cations) are needed to keep an electronic neutral state. Doping and de-doping states can be switched reversibly by applying voltage. Both states are bistable with open-circuit.

Band gap energy of quinoidal structure is smaller than that of aromatic structure. When band gap energy is small enough, the absorption band of CP is shifted to the near-infrared area and there is no absorption band in the visible area (see Figure 1).

At this time, human eyes cannot recognize coloration. Namely, CP has coloration in aromatic structure (de-doping state), meanwhile CP is transparent in quinoidal structure (doping state). This switching can be controlled by applied voltage.

A coloration of CP depends on de-doping level. In other words, full de-doping state has deep color, meanwhile half de-doping state has comparatively pale color. The extent of Doping/de-doping can be controlled by the strength of voltage. The color gradation can be controlled by applied voltage.

**Figure 2:**
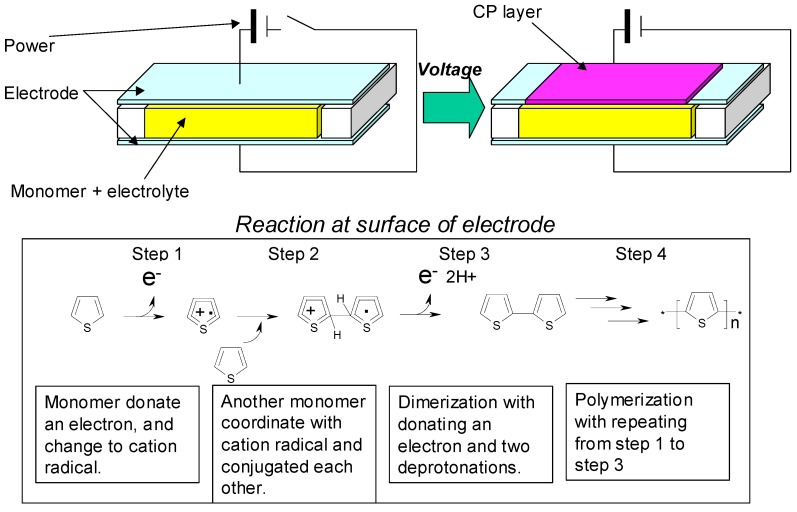
Mechanism of conductive polymer (CP)’s polymerization on electrode.

An electrochemical polymerization is another feature in CP. In electrochemical polymerization of CP, monomers are polymerized at the surface of electrode. For example, in EC type e-paper, the electrode corresponds to ITO. The mechanism of CP’s polymerization on the electrode is shown in Figure 2.

First, a monomer donates an electron to the electrode, and becomes a cation radical (step 1). As a second step, another monomer coordinates with the cation radical. Then they conjugated each other (step 2). As a third step, the conjugated radical dimer donates an electron to the electrode and releases two protons as hydrogen (step 3). An electrochemical polymerization proceeds by repeating from step 1 to step 3.

Since this polymerization needs the electron donation toward the electrode, it happens only on the electrode. Once polymerization proceeds to some extent, CP is unable to be dissolved to any solvent without modifications (basically, monomers can be dissolved to a great variety of solutions). So CPs are piled up onto the electrode. Thus, electrochemical polymerization can be useful for preparing sedimentary EC layer on the electrode like Indium Tin Oxide (ITO). 

## 2. Results and Discussion 

**Figure 3:**
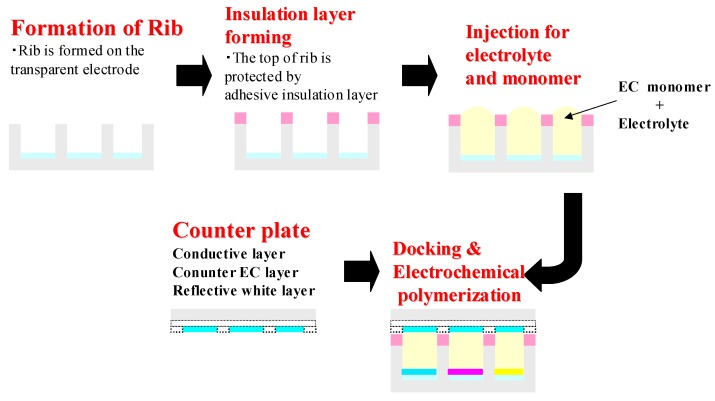
A fabrication process for electrochromic (EC) polymer type e-paper.

In Figure 3, we show our concept of a fabrication process for EC polymer type e-paper. In this fabrication process, the monomer solution dissolved in organic solvent with electrolyte is printed on the electrode by a general printing method such as ink-jet printing, and then sandwiched with the counter electrode such as ITO, then applied appropriate voltage between the electrodes. Once voltage is applied, electrochemical polymerization begins, and finally CP film is made on the surface of the electrode. 

This CP film can be used as an EC layer as it is. Certainly, chemically polymerized CPs are applicable for the printing method [10], in case they are modified with alkyl chains having appropriate length. However there will be some difficulties to maintain the good balance between color variations of EC and solubility. Furthermore, the number of applicable solvent will be limited. That is why electrochemical polymerization is suitable for the fabrication process of printable electronics. 

**Figure 4:**
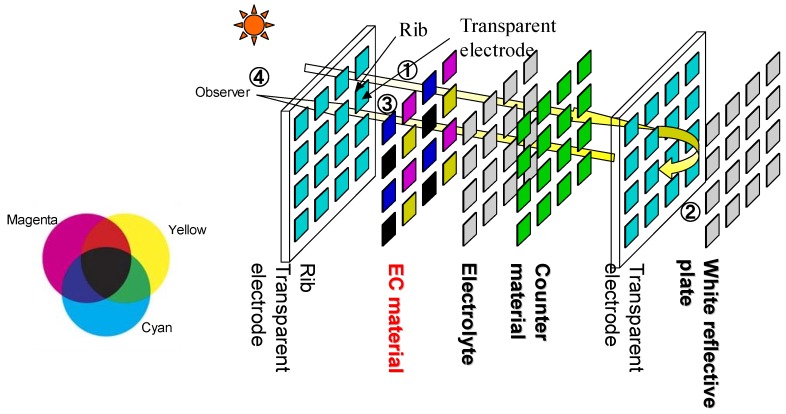
A structure example of EC display.

A structure example of EC display is shown in Figure 4. In this example, an incident light goes through EC layer, then it is reflected at the white reflection plate, and then it goes back again through the EC layer. When the EC layer has coloration, the observer can see certain color, for example cyan, magenta, yellow, and so on. When the EC layer is transparent, the observer can see the white color on the back plane through the EC layer. The EC layer can be applied for both a juxtaposition mixture method and a lamination mixture method.

Here we introduce novel polythiophene derivatives, poly(1H-thieno[3,4-d]imidazol-2(3H)-one) pTIO. These derivatives can change those colors from Cyan, Magenta, or Yellow to transparent. In order to estimate the color of EC in pTIO derivatives, we made use of quantum simulations based on density functional theory. 

**Figure 5:**
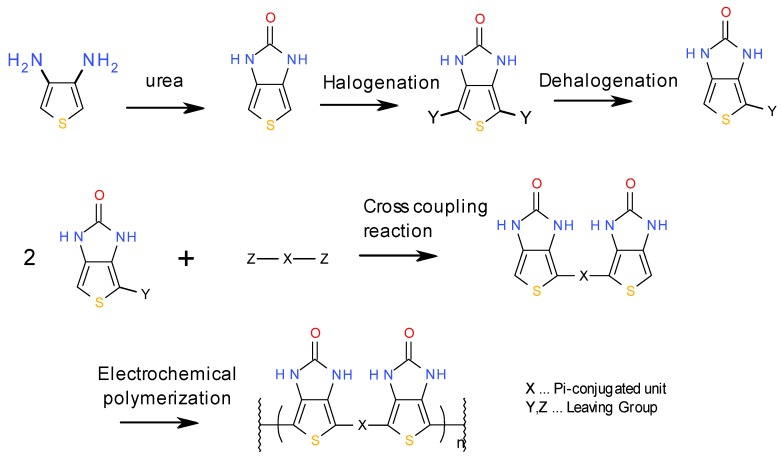
The preparation scheme of our EC polymer.

The representative synthetic route of pTIO derivatives is shown in Figure 5. The color of EC polymers in de-doping state can be adjusted by changing the X unit. So the molecular design was easily done by quantum simulations, as mentioned above. It is preferable for the X unit to be of a pi-conjugated structure. As one example for Magenta, benzene derivatives can be used for the X unit. In the same way, the derivatives of carbazole and thiophene can be used for Yellow and Cyan. 

Since pTIO derivatives have intramolecular hydrogen bond, they have high planer polymer backbone. These trimers have good solubility into various solutions with electrolytes. The solution containing trimers and electrolytes is suitable for printed electronics because the liquid properties can be adjusted easily, which is important for printing methods. 

Electrochromism of these polymers shows the color change between coloration and pale gray. These color changes can be observed at low applied voltage (+1.5 V ~ −0.5 V *vs*. Ag/AgCl reference). Here, we define the response time which takes a certain time length to change its color from the full-doping state to the de-doping state under the applied constant current. The response times of these pTIO were approximately 500 ms. At least 4 gradations can be reproduced depending on applied voltage.

**Figure 6:**
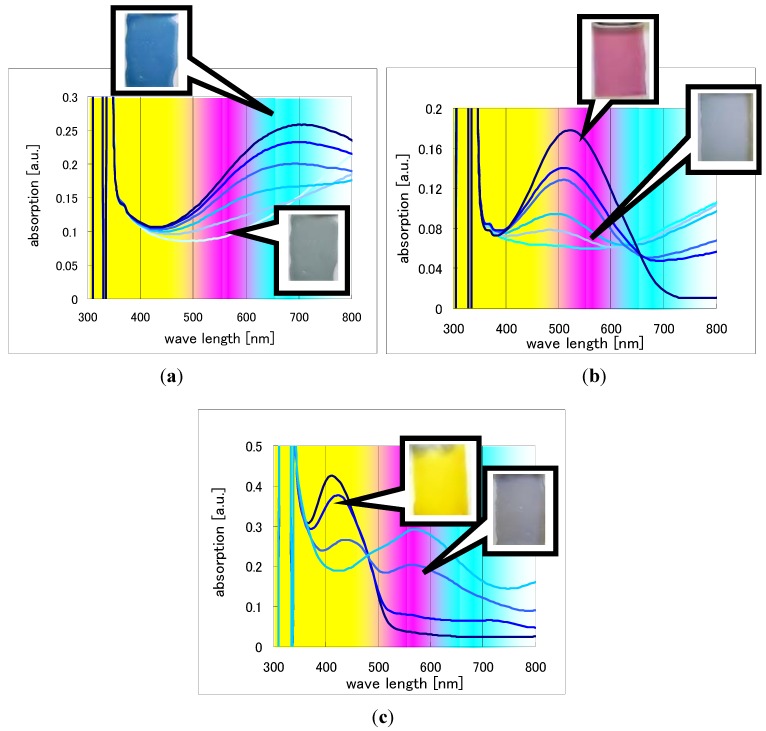
UV-Vis spectra of each color (**a**) Cyan; (**b**) Magenta; and (**c**) Yellow.

We show the UV-Vis spectra of each EC polymer, (a) Cyan; (b) Magenta; and (c) Yellow in Figure 6. 

These UV-Vis spectra were measured in voltage range from +1.5 V to −0.5 V (*vs*. Ag/AgCl reference). Applied voltage was increased by each 0.2 V step in this measurement. Since at some applied voltages obvious changes were not observed in UV-Vis spectra, these were omitted from the graphs. In case of Cyan and Magenta, six gradations were observed, meanwhile only four gradations were observed in Yellow. In addition, its doping state was slightly bluish.

As EC polymer in this Yellow color has higher band gap energy than other colors’, which can show us yellow color, the extent of band gap energy change between doping and de-doping needs to be larger than other colors in order to be transparent in the doping state. In this case, the extent of energy change was not enough large. That is why the doping state was bluish. Another X unit needs to be developed in the future.

**Figure 7:**
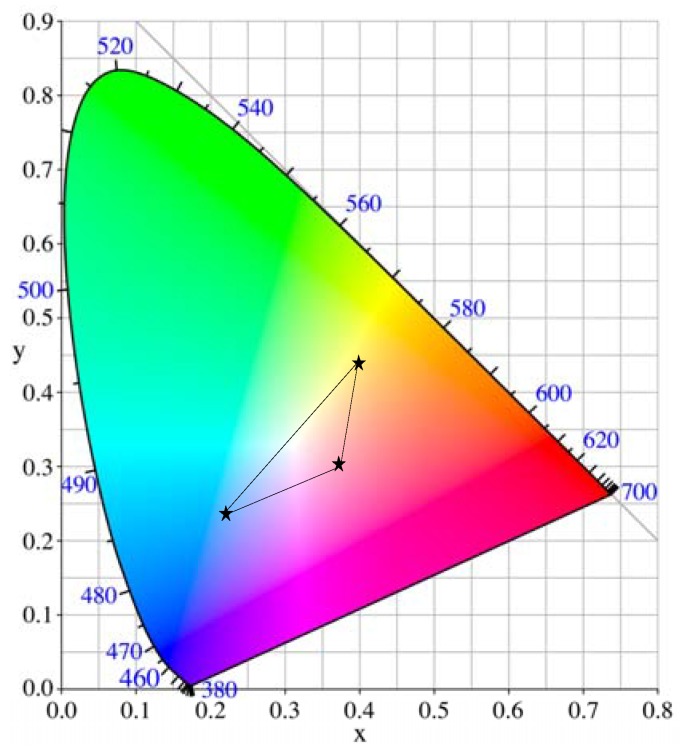
Color image on CIExy 1931.

We show the color images of these EC polymers in fully de-doping state on CIExy 1931 in Figure 7. These colors are well adjusted by changing X unit except for the Magenta color.

**Figure 8:**
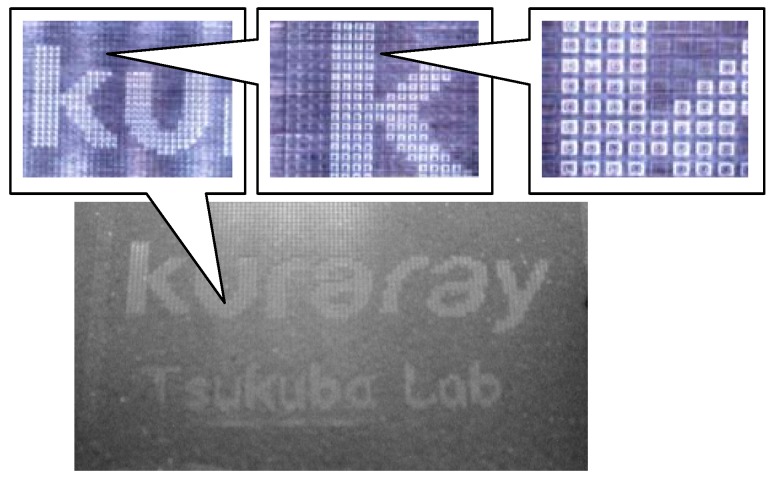
The image of enlarged image of electrochromic display (ECD).

**Figure 9:**
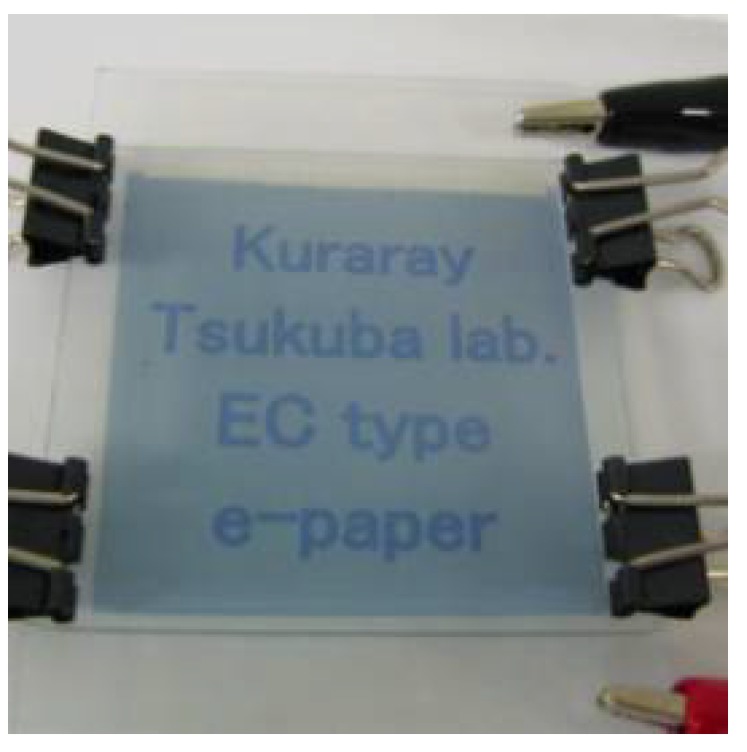
The image of ECD.

The images of mono-color segment matrix type display are shown in Figure 8 (the image of an enlarged image of ECD) and Figure 9 (the image of ECD). These electrochromic displays (ECD) were fabricated by using ink-jet printing as follows. 

The fabrication process by ink-jet printing was tried as follows.

1. Prepared squared rib (50 × 50 um/one grid ) which was formed by photolithography on ITO glass electrode.
2. Dissolved monomers as precursors of EC polymers into organic solvent such as propylene carbonate. Added supporting electrolyte such as tetra butyl ammonium perchlorate into the solution. By choosing appropriate solvents, liquid properties were optimized for printing process.
3. Poured the solution above into each cite by ink-jet printing.
4. Glued with another ITO glass electrode.
5. Applied certain voltage (for example +1.5 V) to each site for proper time (for example 10–60 s). EC polymer layer was formed on the ITO electrode.
6. Switched applied voltage (for example −0.5 V), the color of EC polymer layer was changed from coloration to transparent.

These ECD consisted of two 5 × 5 cm ITO glass electrodes and rib. The characters on these segment matrix displays can switch between transparent and blue coloration. 

**Figure 10:**
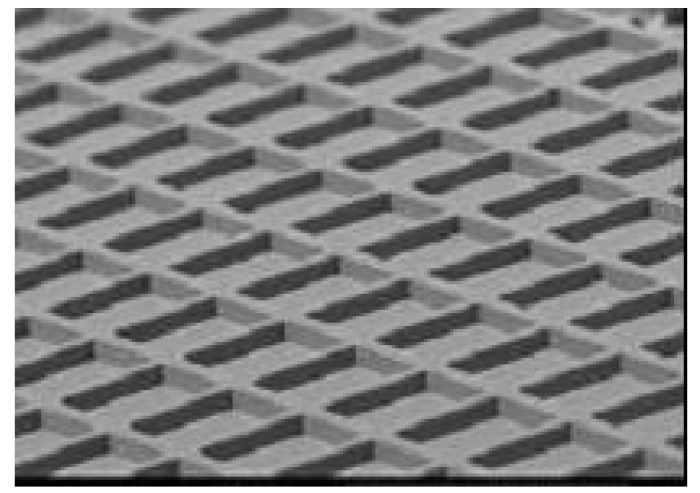
The image of rib on Indium Tin Oxide (ITO) glass.

**Figure 11:**
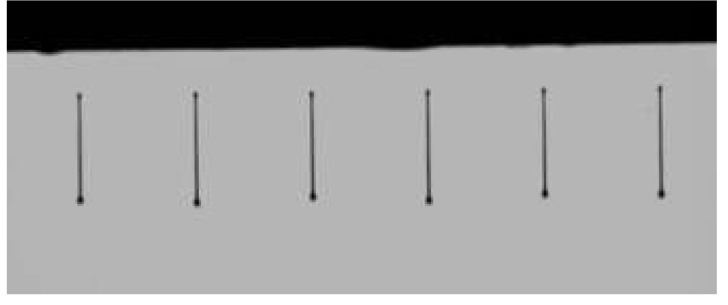
The image of ink-jet printing.

In Figure 10, we show the image of rib on ITO glass, and in Figure 11 the image of ink-jet printing is shown. 

A rib formed by photolithography on the ITO glass electrode is separating adjacent pixels (see Figure 10). Although we used rigid substrates in this work, it is possible to substitute flexible substrates such as ITO coated films for the rigid ones. Since the solution properties such as surface tension, viscosity, and boiling point were well adjusted for ink-jet printing, straight liquid pillars forward substrates were able to be observed (see Figure 11).

## 3. Experimental Section 

### 3.1. Synthesis for 3,4-Diaminothiophene

3,4-diaminothiophene dihydrochloride was dissolved in water (4 mL/mmol), and a 4 N aqueous sodium carbonate solution (2 mL/mmol) was dropped slowly, followed by stirring for additional 2 h after the dropping. The product was extracted to an organic layer using ethyl acetate and the resulting organic layer was dried over sodium sulfate and then the solvent was distilled off, so that 3,4-diaminothiophene was obtained (yield: 60%).

^1^H-NMR (500 MHz, CDCl_3_, TMS) δ: 6.17 (2H, s), 3.36 (4H, s).

### 3.2. Synthesis for 1H-Thieno[3,4-d]Imidazol-2(3H)-One 

3,4-diaminothiophene and urea (1.1 eq.) were added into amyl alcohol (10 mL/mol). The reaction was advanced under reflux at 130 °C for 5 h in inert atmosphere, and then the amyl alcohol was evaporated, followed by a purification step by using column separation with an ethyl acetate/hexane solvent, so that 1H-thieno [3,4-d]imidazol-2(3H)-one was obtained (yield: 60%).

^1^H-NMR (500 MHz, CDCl_3_, TMS) δ: 6.36 (1H, s).

### 3.3. Synthesis for 4,6-Dibromo-1H-Thieno[3,4-d]Imidazol-2(3H)-One

1H-thieno[3,4-d]imidazol-2(3H)-one was dissolved in tetrahydrofuran (10 mL/mmol) and was kept at −78 °C in a dry ice-cooled methanol bath. To this was dropped slowly 2.1 equivalents of N-bromosuccinic imide dissolved in tetrahydrofuran of an amount of 5 mL/mmol relative to the 1H-thieno[3,4-d]imidazol-2(3H)-one, followed by a reaction for 30 min, and then the reaction was stopped by adding an excessive amount of a saturated aqueous sodium chloride solution. The product was extracted from the reaction liquid to an organic layer using diethyl ether and dried over sodium sulfate and then the solvent was evaporated, followed by a purification step by column separation using an ethyl acetate/hexane solvent, so that 4,6-dibromo-1H-thieno[3,4-d]imidazol-2(3H)-one was obtained (yield: 90%).

^1^H-NMR (500 MHz, DMSO, TMS) δ: 11.01 (2H, s).

### 3.4. Synthesis for 4-Bromo-1H-Thieno[3,4-d]Imidazol-2(3H)One

4,6-dibromo-1H-thieno[3,4-d]imidazol-2(3H)-one was dissolved in of dry tetrahydrofuran (10 mL/mmol) and was kept at −78 °C in a dry ice-cooled methanol bath. Under inert gas atmosphere, a 1.6 N n-butyl lithium/hexane solution in an amount of 3.1 equivalents relative to 4,6-dibromo-1H-thieno[3,4-d]imidazol-2(3H)-one was dropped slowly, followed by a reaction for 15 min, and then the reaction was stopped by the addition of 5 equivalents of 1N hydrochloric acid. The product was extracted from the reaction liquid to an organic layer using diethyl ether and dried over sodium sulfate and then the solvent was evaporated, followed by a purification step by column separation using an ethyl acetate/hexane solvent, so that 4-bromo-1H-thieno[3,4-d]imidazol-2(3H)one was obtained (yield: 60%).

^1^H-NMR (500 MHz, DMSO, TMS) δ: 6.51 (1H, s), 10.50 (1H, s), 10.77 (1H, s).

### 3.5. Synthesis for 1,4-ditributyltinbenzene

1,4-Dibromobenzene was dissolved in dry tetrahydrofuran (2 mL/mmol) and was kept at −78 °C in a dry ice-cooled methanol bath. Under inert gas atmosphere, a 1.6 N n-butyl lithium/hexane solution in an amount of 1.1 equivalents relative to 1,4-dibromobenzene was dropped slowly, followed by a reaction for 30 min, and then 1.0 equivalent of tributyl tin chloride was added, followed by a reaction for 1 hour. Moreover, a 1.6 N n-butyl lithium/hexane solution in an amount of 1.1 equivalents relative to 1,4-dibromobenzene was dropped slowly, followed by a reaction for 30 min, and then 1.0 equivalent of tributyl tin chloride was added, followed by a reaction for 1 hour, and then the reaction was stopped by the addition of an excessive amount of a saturated aqueous sodium chloride solution. Washing was done three times using a saturated aqueous sodium chloride solution, then the product was separately extracted from the resulting reaction liquid to an organic layer using diethyl ether, followed by drying over sodium sulfate, and then the solvent was evaporated, so that 1,4-ditributyltinbenzene was obtained.

^1^H-NMR (500 MHz, CDCl_3_, TMS) δ: 7.40 (4H, s), 1.54 (12H, quint.), 1.32 (12H, h), 1.03 (12H, t), 0.88 (18H, t).

### 3.6. Synthesis for 4-[4-(2-oxo-2,3-dihydro-1H-thieno[3,4-d]imidazol-4-yl)phenyl]-1H-thieno[3,4-d]imidazol-2(3H)-One

To 1,4-ditributyltinbenzene were added 2.0 equivalents of 4-bromo-1H-thieno[3,4-d]imidazol-2(3H)-one, 5 mL/mol of dry toluene, and 0.2 equivalents of trans-dichloro bis-triphenyl phosphine palladium, then the reaction was advanced under reflux at 130 °C for 40 h in inert gas atmosphere, and then the reaction was stopped by the addition of a saturated aqueous ammonium chloride solution. The product was extracted from the resulting reaction liquid to an organic layer using diethyl ether, followed by drying over sodium sulfate, and then the solvent was evaporated, followed by a purification step by column separation using an ethyl acetate/hexane solvent, so that 4-[4-(2-oxo-2,3-dihydro-1H-thieno[3,4-d]imidazol-4-yl)phenyl]-1H-thieno[3,4-d]imidazol-2(3H)-one was obtained.

^1^H-NMR (500 MHz, CDCl_3_, TMS) δ: 6.33 (2H, s), 7.44 (4H, s).

## 4. Conclusions 

In this paper, our novel EC polymer and fabrication method of EC type segment matrix e-paper are introduced. 

Electrochromism of CP and electrochemical polymerization from its corresponding mononers are applicable for printed electronics. We developed novel EC polymers, pTIO derivatives the color of which change from Cyan, Magenta, and Yellow to clear (slightly pale gray). We also fabricated ECD with these novel EC polymers. 

Our concept of preparation for various colors is just changing X unit in co-polymerization. We prepared corresponding trimer prior to electrochemical polymerization, which make it possible to apply ink-jet printing method. We adjusted the colors of Cyan, Magenta and Yellow by electrochemical polymerization of those trimers. The gradation of EC polymers were well-controlled depending on applied voltages. Applied voltages are basically low, which enable to drive ECD below +/−1.5 V. In addition, our EC polymers are suitable for see-through type display, because pTIO can change its color to clear. 

However, there are still some problems. First, the contrast between coloration and transparency needs to be improved. Second, the performance of mixed color needs to be adjusted more. Third, the cross-talk problem in passive-matrix driving has to be solved. Forth, the optimization of device structures is necessary, which leads to the enhancement of memory effect and cyclability in EC device. For example, the active materials on the counter electrode will be required in order to compensate for the complementary RedOx reaction against the EC materials. 
